# The Influence of the Coexpression of CD4 and CD8 in Cutaneous Lesions on Prognosis of Mycosis Fungoides: A Preliminary Study

**DOI:** 10.1155/2014/624143

**Published:** 2014-07-20

**Authors:** Sergio Umberto De Marchi, Giuseppe Stinco, Enzo Errichetti, Serena Bonin, Nicola di Meo, Giusto Trevisan

**Affiliations:** ^1^Institute of Dermatology and Venereology, University of Trieste, Ospedale Maggiore, Piazza Ospedale 1, 34100 Trieste, Italy; ^2^Institute of Dermatology, Department of Experimental and Clinical Medicine, University of Udine, Ospedale San Michele, Piazza Rodolone 1, 33013 Gemona del Friuli, Italy

## Abstract

*Background*. Although techniques of immunophenotyping have been successful in characterizing the cells in the cutaneous infiltrates of mycosis fungoides little evidence suggests that variations in the phenotypic characterization correlate with prognosis. *Objectives*. In a preliminary prospective, single-centre, study we correlated the T-cell phenotype in cutaneous biopsies with the progression of the disease to determine whether the coexpression of CD4 and CD8 has an impact on prognosis. *Methods*. Skin biopsy specimens from 30 newly diagnosed patients were stained with immunoperoxidase techniques to determine their phenotypic characteristics. After a median followup of 42 months patients were divided into two groups with stable and progressive disease. *Results*. Eighteen patients had the conventional CD4+CD8− T-cell phenotype. Ten patients showed the coexpression of CD4 and CD8 and had a slightly lower rate of progressive disease. *Conclusions*. The coexpression of CD4 and CD8 in cutaneous lesions is not rare and is associated with a slightly lower rate of progressive disease. Since double positive CD4/CD8 phenotype is rarely reported in mycosis fungoides the presence on conventional immunophenotyping of both CD may be due to a “mixture” of neoplastic cells and inflammatory CD8+ tumor infiltrating lymphocytes. Immunohistochemical study combined with confocal microscopy could clarify this issue.

## 1. Introduction

Mycosis fungoides (MF) represents the prototype of cutaneous T-cell lymphoma, which is defined as clonal expansion of skin-homing T lymphocytes [1]. The natural history of MF is characterized by an indolent progression through four stages: patch, plaque, tumor, and visceral involvement. The disease begins with lightly erythematous patches that subsequently evolve into well-demarcated scaling plaques. These plaques may then progress to tumor lesions and subsequently spread to the viscera, but this progression is not necessarily seen in all patients [2]. MF is classified into clinical stages using the TNM classification. Previous studies of prognostic indicators have shown that the skin (T) stage and the presence or absence of extracutaneous disease are the most important determinants of outcome. Patients with limited skin involvement (T1) have a favorable prognosis, whereas patients with tumor (T3) or erythrodermic MF (T4) have an unfavorable prognosis [3, 4]. The diagnosis of MF relies on histopathological examination of skin biopsies [5]. Usually, MF is characterized by an infiltrate of *α*/*β* T helper memory lymphocytes (*β*F1+, CD3+, CD4+, CD5+, CD8−, and CD45RO+) [1]. However, in a minority of cases the neoplastic cells exhibit a T-cytotoxic (CD4−CD8+), *γ*/*δ* (*β*F1−, CD3+, CD4−, CD5+, and CD8+) or a CD4/CD8 double-negative phenotype, that show no clinical and/or prognostic differences [6]. Recently, a previously unrecognized phenotype characterized by coexpression of CD4 and CD8 has been described [7, 8], but the influence of such a phenotype on prognosis of MF has not been evaluated. Therefore, although techniques of immunophenotyping have been successful in characterizing the cells in the cutaneous infiltrates of MF little evidence suggests that variations in the phenotypic characterization correlate with prognosis.

In this preliminary prospective, single-centre study on a limited number of patients with MF we correlated the T-cell phenotype in cutaneous lesions with the progression of the disease to determine whether the coexpression of CD4 and CD8, compared to conventional CD4+CD8− phenotype, has an impact on prognosis.

## 2. Patients and Methods

Patients with MF were prospectively included in this study between January 2005 and December 2012, after giving informed consent according to the declaration of Helsinki. The diagnosis of MF was made according to the criteria of the WHO-EORTC classification for cutaneous lymphomas [1]. The clinical stage at presentation was determined using the TNM classification adapted for MF [9]. Patients received a comprehensive history and physical examination, complete blood cell count including peripheral smear for Sézary cells, and general chemistry panel. Lesional cutaneous biopsies were obtained in all patients upon admission into the study before the start of therapy. Patients with clinically significant adenopathy had their nodes evaluated by fine needle aspiration or lymph node biopsy. When indicated, patient had an extensive staging evaluation, including bone marrow aspirate and biopsy, and appropriate radiologic studies to determine visceral involvement.

We included in the study only newly diagnosed patients who had not previously received topical therapies (corticosteroids, topical nitrogen mustard, carmustine, and psoralen with ultraviolet A), interferon, chlorambucil, methotrexate, or polychemotherapy.

After initial evaluation, the patients were included into a program of visits that were made every three months throughout the period of followup. According to the course of the disease and, more specifically, to their clinical status at the time of the last clinical update, patients were classified into two groups. The first group was formed by patients with a progressive disease defined as the change to a more advanced stage. In the second group patients who underwent complete or partial remission and patients with stable disease defined as presence of MF without progression were included. Factors associated with disease progression were studied with special emphasis to T-cell phenotype. The study did not consider the therapy received by the patients. Indeed treatments were administered according to clinical stage and were relatively homogeneous within each study group for most of the patients, but no prospective therapeutic trial was simultaneously performed. At early stages (I-IIa), most of the patients were treated by topical therapies (corticosteroids, topical nitrogen mustard, carmustine, and psoralen with ultraviolet A). At advanced stages, radiotherapy was performed on tumors, and interferon, chlorambucil, methotrexate, or polychemotherapy was administered as needed.

## 3. Histopathologic and Immunohistologic Analysis

Formalin fixed and paraffin-embedded tissues of lesional cutaneous biopsies were obtained from all patients. Specimens were fixed in 10% buffered formalin and subsequently embedded in paraffin. Sections were stained with haematoxylin and eosin for routine histopathological evaluation. The diagnosis of MF was established according to the criteria proposed by Guitart et al. [10].

Immunostaining was performed on fixed, paraffin-embedded tissue sections as previously described [11] using monoclonal antibodies specific for T-cell associated antigens (CD3, CD4, and CD8) and for B cell-associated antigens (CD20).

## 4. Statistical Analysis

Statistical analysis was performed with dedicated STATA SE 12 (Stata Corporation, TX, USA). The Fisher's exact test and the Pearson *χ* ^2^-test were used to identify the differences between the variables. A multivariate logistic regression was used to evaluate the relationship among variables. Logistic regression is a variation of ordinary regression which predicts the probability of the occurrence of a specific event as a function of two or more independent variables. The results obtained were expressed in terms of the odds ratio (OR) associated with each predictor value, defined as the probability of the event occurring divided by the probability of the event not occurring. In all analyses, the cut-off level of statistical significance was set at 0.05.

## 5. Results

Thirty patients (17 men and 13 women) with clinical and histologic diagnosis of MF were included in the study. The mean age at diagnosis was 64.5 years (range 49–78 years). According to the TNM classification 8 patients were in stage I, 13 in stage II, 6 in stage III, and 3 in stage IV. The thirteen patients in stage II were classified as 6 stage IIa and 7 stage IIb. Stages I-IIa were considered as early stages and stages IIb–IV as advanced stages. At the time of diagnosis, MF was in a more advanced stage in women than men. Indeed, 52.9% of men (versus 38.4% of women) were in early stages whereas 61.6% of women (versus 47.1% of men) were in advanced stages.

Immunophenotypic analysis demonstrated that 93.3% (28 out 30) of the patients with MF were CD4 positive. CD20 was negative in all patients. Eighteen out 30 patients had the mature CD4+/CD8− T-cell phenotype. The cytotoxic T-cell phenotype CD4−/CD8+ was present in only one patient as well as the CD4/CD8 double-negative phenotype. Ten out of 30 patients (33.3%) had an immunophenotypic profile characterized by the coexpression of CD4 and CD8. When we compared the distribution of patients with the coexpression and those with the conventional CD4+/CD8− phenotype according to the TNM staging we could not find any association between phenotypes and clinical stage. Indeed the percentages of the patients with advanced stage, as compared with those in early stage, were not significantly different in both groups (group CD4+/CD8− 50% versus 50%; group CD4+/CD8+ 40% versus 60%).

The median follow-up time in the study was 42 months (ranging from 12 to 70 months) and was comparable among the different clinical stages and phenotypes. None of the patients died during the study period. At the end of the followup 23 of 30 patients underwent complete or partial remission or presented a stable disease without extension of the lesions or increase of the number of lesions. Seven patients showed a progressive disease defined as the change to a more advanced stage. The age of patient at diagnosis (<65 years versus >65 years), the sex, the duration of symptoms before diagnosis of MF (<60 months versus >60 months), and the TNM stage at presentation (early stages versus advanced stages) were not associated with disease progression in univariate as in multivariate analysis. The results of the logistic regression analysis are reported in Table 1. None of the independent variables introduced in the logistic regression model was associated with disease progression defined as the change to a more advanced stage. However, by analyzing the distribution of T-cell phenotypes within the progressing and nonprogressing groups we found that patients with the coexpression CD4 and CD8 had a slightly lower rate of progressive disease as compared to patients with conventional phenotype (10.0% versus 27.8%). In the logistic regression model the value obtained by the independent variable phenotype has almost reached the statistical significance (odds ratio 17.03, *P* = 0.09, 95% confidence interval 0.62–467.7).

## 6. Discussion

In this preliminary prospective, single-centre study, we found by conventional immunophenotyping a profile characterized by the coexpression of CD4 and CD8 in one-third of patients with MF. These patients showed a slightly lower rate of progressive disease, compared to patients with conventional CD4+/CD8− phenotype. These findings raise the possibility that the coexpression of CD4 and CD8 in cutaneous lesions may confer a better prognosis in MF.

In our patients the conventional T-helper phenotype (CD4+/CD8−) was the most common (60% of patients), thus confirming previous studies [1, 12]. Twelve patients had a different phenotype. Two patients showed, respectively, the cytotoxic phenotype (CD4−/CD8+) and the double negative phenotype (CD4−/CD8−). The conventional immunostaining in the remaining 10 patients (33.3%) showed the coexpression of CD4 and CD8.

The coexpression of CD4 and CD8 is an expected event on common thymocytes, but it is fairly infrequent on normal peripheral T lymphocytes. There is evidence that in certain situations the normal CD4+ cells may coexpress CD8 and interleukin-4 is able to induce the expression of CD8 on T CD4+ clones [13]. Peripheral T lymphocytes with CD4+/CD8+ phenotype have been described in some solid and hematologic malignancies [14, 15]. However the double positive CD4/CD8 phenotype is extremely rare in mycosis fungoides [7, 8]. The findings of our patients may not be due to the coexpression of both CD in the same neoplastic cell but to the presence in the cutaneous lesions of a “mixture” of neoplastic cells and inflammatory CD8+ tumor infiltrating lymphocytes as previously demonstrated by Hoppe et al. [16]. Immunohistochemical study combined with confocal microscopy might clarify this issue as recently reported by Tournier and coworkers [8] in a 31-year-old woman. In their patient this technique revealed in lesional cutaneous biopsies the coexpression of CD4 and CD8 in a subset of atypical T lymphocytes.

In typical lesional biopsy specimens of MF other nonneoplastic mononuclear cells are present including CD8+ tumor-infiltrating lymphocytes. The role that these cells play in etiopathogenesis and natural history of MF is still unclear [17].

In our patients with MF the subgroup with the coexpression of CD4 and CD8 has a slightly lower rate of progressive disease in comparison to patients with conventional CD4+/CD8− phenotype (10.0% versus 27.8%), and in the logistic regression model the value obtained by the independent variable T-cell phenotype has almost reached the statistical significance.

This data might be of interest taking into account that the limited number of patients and the median follow-up period of just 3.5 years, although similar to those of other investigations, may not allow adequate time for this indolent lymphoma to progress.

The lower tendency to progression of disease in our patients with the coexpression of CD4 and CD8 might be related to increased activity of antitumor CD8+ lymphocytes infiltrating the lesions. Our results may be consistent with previous findings by Hoppe et al. [16] who demonstrated that CD8+ tumor-infiltrating lymphocytes influenced the long-term survival of patients with MF.

Another problem is the possibility that CD8+ inflammatory cells can appear in the course of treatment. This is not the case of our patients because we have included in the present study only newly diagnosed patients who had not previously received topical therapies (corticosteroids, topical nitrogen mustard, carmustine, and psoralen with ultraviolet A), interferon, chlorambucil, methotrexate, or polychemotherapy.

Finally, in this preliminary prospective, single-centre study, conventional immunophenotyping demonstrates the coexpression of CD4 and CD8 in lesional cutaneous biopsies of one-third of patients with MF. These patients show a slightly lower rate of progressive disease, compared to patients with conventional CD4+/CD8− phenotype. A prospective multicenter study on a larger population of patients with a longer followup might be useful to confirm if the coexpression of CD4 and CD8 may confer a better prognosis in patients with MF.

## Figures and Tables

**Table 1:** Factors associated with the disease progression in patients with mycosis fungoides.

Disease progression	Odds ratio	Standard error	*z*	*P* value	95% confidence interval	
Phenotype	17,03	28,79	1,68	0,09	0,62	467,7
TNM stage	2,07	1,19	1,26	0,21	0,67	6,39
Duration of followup	1,19	0,16	1,31	0,19	0,91	1,55
Age at diagnosis	0,29	0,38	0,94	0,35	0,02	3,82

In the logistic regression model the dependent variable was the disease progression defined as the change to a more advanced stage; the independent variables were T-cell phenotype (CD4+CD8+ vs CD4+CD8−), TNM stage at presentation, duration of followup, and age at diagnosis.
